# Brazilian version of the Frontal Assessment Battery (FAB): Preliminary data on administration to healthy elderly

**DOI:** 10.1590/S1980-57642008DN10100010

**Published:** 2007

**Authors:** Rogério Gomes Beato, Ricardo Nitrini, Ana Paula Formigoni, Paulo Caramelli

**Affiliations:** 1 MD, MSc, Behavioral and Cognitive Neurology Unit, Faculty of Medicine, Federal University of Minas Gerais, Belo Horizonte, Brazil.; 2 MD, PhD, Behavioral and Cognitive Neurology Unit, Department of Neurology, and Cognitive Disorders Reference Center (CEREDIC). Hospital das Clínicas of the University of São Paulo School of Medicine, São Paulo, Brazil.; 3 PhD, Behavioral and Cognitive Neurology Unit, University of São Paulo School of Medicine, São Paulo, Brazil.; 4 MD, PhD, Behavioral and Cognitive Neurology Unit Faculty of Medicine, Federal University of Minas Gerais.

**Keywords:** frontal lobe, prefrontal cortex, aging, education, neuropsychological tests, lobo frontal, córtex pré-frontal, envelhecimento, educação, testes neuropsicológicos

## Abstract

The Frontal Assessment Battery (FAB) has been proposed as a diagnostic tool for patients with frontal lobe syndrome.

**Objectives:**

To present the Brazilian version of the FAB and to show preliminary data on the performance of healthy elderly in the battery, correlating with age, education and scores in the Mini- Mental State Examination (MMSE).

**Methods:**

Forty-eight healthy elderly individuals (34 female/14 male) were evaluated, aged 69.3±6.1 years and with educational level=8.0±5.6 years. The subjects were submitted to the MMSE, the Cornell depression scale and the FAB, in which scores were determined for each item and for the total scale. All individuals had to attain above education adjusted cut-off scores in the MMSE and =7 points on the Cornell depression scale. Correlations were calculated between FAB total scores and age, educational level and MMSE scores, as well as between FAB items and education.

**Results:**

The mean score ±SD in the FAB was 13.0±2.3(7 to 18). Total FAB scores correlated significantly with education (r=0.37; p=0.01) and MMSE scores (r=0.46; p=0.001). No correlation emerged between FAB scores and age. The mean score ±SD of the MMSE was 27.4 ± 1.8. Considering the six FAB items separately, two of them (similarities and conflicting instructions) correlated significantly with educational.

**Conclusions:**

In this group of healthy elderly, the Brazilian version of the FAB proved to be influenced by education, but not age.

## Abstract (pt)

A Bateria de Avaliação Frontal (BAF) foi proposta recentemente como instrumento diagnóstico para pacientes com síndrome frontal.

**Objetivos:**

Apresentar a versão brasileira da BAF e dados preliminares do desempenho de idosos saudáveis na bateria, e sua correlação com a idade, nível educacional e escores no Mini- Exame do Estado Mental (MEEM).

**Métodos:**

Foram avaliados 48 idosos saudáveis (34 mulheres/14 homens), com idade média=69,3±6,1 anos e escolaridade média=8,0±5,6 anos. Todos foram submetidos ao MEEM, à escala de Depressão de Cornell e à BAF, com escores determinados para cada item e no total. Os participantes apresentaram desempenho acima de valores ajustados para a escolaridade no MEEM e =7 pontos na escala de Depressão de Cornell. Foram calculadas correlações entre o escore total da BAF e as variáveis idade, escolaridade e escore do MEEM, como também a correlação entre os itens da BAF e a escolaridade.

**Resultados:**

O escore médio ±DP na BAF foi 13,0±2,3 (7-18). Os escores totais da BAF se correlacionaram significativamente com a escolaridade (r=0,37; p=0,01) e com os escores do MEEM (r=0,46; p=0,001). Não foi observada correlação entre a BAF e a idade. O escore médio do MEEM foi 27,4 ± 1,8. A análise separada dos itens da BAF mostrou que dois deles (similaridades e instruções conflitantes) se correlacionaram significativamente com a escolaridade.

**Conclusões:**

Neste grupo de idosos saudáveis, a versão brasileira da BAF demonstrou ser influenciada pela escolaridade, mas não pela idade.

Executive functions are mental processes involved in the realization of goal-directed behavior whether expressed through a mental or a motor act. They are thought to control formulation, planning, carrying out and effective performance of goal-oriented actions^1^. Executive functions are frequently impaired after frontal lobe or basal ganglia damage. In general, evaluation of executive functions is performed with time-consuming neuropsychological tests.

The Frontal Assessment Battery (FAB) has been proposed recently as a brief diagnostic tool to be used in cases of disexecutive syndrome^2^. It can be performed in approximately ten minutes. The FAB has been used in several groups of patients, such as Alzheimer’s disease^3,4^, frontotemporal dementia^3,4^, Parkinson’s disease^5^, atypical parkinsonian syndromes^6^ and vascular focal lesions^7^. The aim of the present study was to evaluate the performance of normal elderly on the FAB, and correlate to age, schooling and score in the Mini-Mental State Examination (MMSE).

## Methods

Individuals were caregivers of demented patients evaluated at the Behavioral and Cognitive Neurology Unit of the Faculty of Medicine of Federal University of Minas Gerais and volunteers recruited from the community.

The inclusion criteria were absence of neurological or psychiatric diseases, absence of depression and no use of benzodiazepines, antidepressants or neuroleptics.

A total of 48 cognitively intact elderly individuals (34 female and 14 male), aged 60 to 91 years (mean±SD= 69.3±6.1), and with educational level ranging from 1 to 20 years (mean±SD=8.0±5.6), were evaluated.

All participants were submitted to the Mini-Mental State-Examination (MMSE), to the Cornell scale of depression and to the FAB, in which scores were determined for each item and for the total scale. Performance in the MMSE adjusted to the educational level, had to be greater than or equal to 21 for 1-3 years of schooling, greater than or equal to 24 for 4-7 years and greater than or equal to 26 for individuals with 8 or more years of schooling^8^. Score on the Cornell scale of depression had to be less than or equal to 7 points in order to rule out depression^9^.

The FAB consists of six subtests:

1. ***Similarities*** – Abstract reasoning is frequently impaired in subjects with frontal lobe lesions^10,11^. Such individuals present difficulties conceptualizing and finding the link between two objects belonging to the same semantic category (e.g. pear and peach)^12^.
2. ***Lexical fluency (letter S)*** – Cognitive flexibility is a broad term used to refer to a person’s ability to switch from one topic to another. To perform this task subjects are required to inhibit one behavior and commence another^13^. Frontal lobe damage, regardless of side, is associated to reduction of verbal fluency^14-17^.
3. ***Motor series*** – To perform a sequence of gestures individuals have to organize, to maintain and to execute successive actions. This task may be impaired in patients with frontal lobe lesions^18-20^.
4. ***Conflicting instructions*** – In this kind of task, as seen in the Stroop test, individuals have to inhibit prepotent stimulus and select the appropriate one^21,22^. Normal subjects are able to follow the examiner’s command and not to do what they see. Subjects with frontal lobe lesions are not able to obey verbal command and tend to imitate the examiner’s gestures^23^.
5. ***Go / No-Go*** – This task requires the subject to make a response to a go signal and withhold the response to no-go signal^24^. Subjects with orbitofrontal lesions are impaired in this kind of task^25-27^.
6. ***Prehension behavior*** – Grasping reflexes are elicited by applying pressure to the palm of the hand^28^. Patients with frontal lobe lesions may present a lack of internal control and are dependent on environmental stimuli^29^. They are sensitive to sensory stimulus and are unable to inhibit the behavior of taking the examiner’s hands^30,31^.

The maximum score for each subtest is 3 points and the total score of test is calculated by adding the scores of the six subtests (maximum score=18).

The FAB was translated from English into Portuguese following a thorough methodology^32^. Initially, translation of the instrument was performed by two independent translators. These two translations were then compared and an initial version in Portuguese was produced. Subsequently, back-translation into English was performed, also by two translators, in order to identify possible discrepancies in the English to Portuguese translation.Minor differences were identified and were discussed by a small panel of specialists. A final consensual Portuguese version was produced and used in the present study. The Brazilian version of the FAB is presented in Appendix.

The total scores of the FAB correlated to the scores of the MMSE, to age and to educational level. In addition, each of the six subtests also correlated to educational level. The normality of the distribution of the total FAB scores was ascertained through the Kolmogorov-Smirnov test. Pearson correlation coefficients were calculated between the different variables of interest. Statistical significance was defined as p values <0.05. Statistical analysis was performed using the MedCalc software.

The study was approved by the Research Ethics Committee of the Federal University of Minas Gerais and all participants signed the approved written informed consent.

## Results

**Graph 1:**
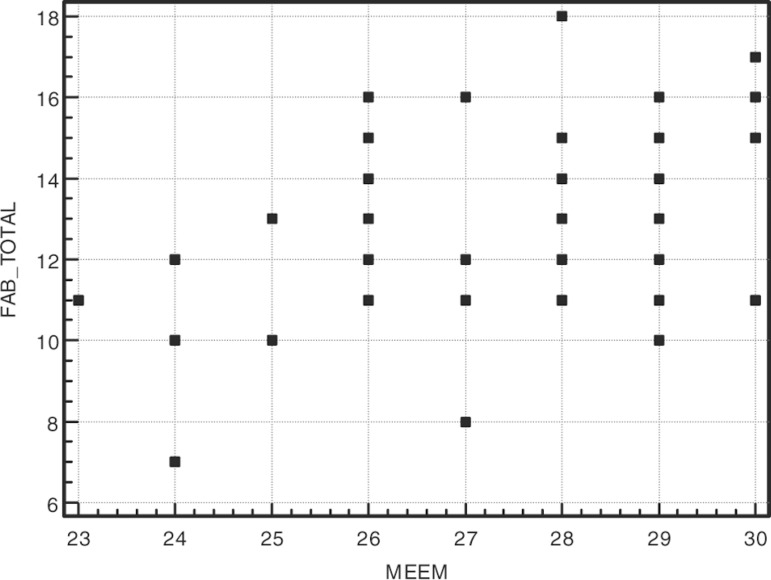
Correlation between FAB total score and MMSE.

**Graph 2:**
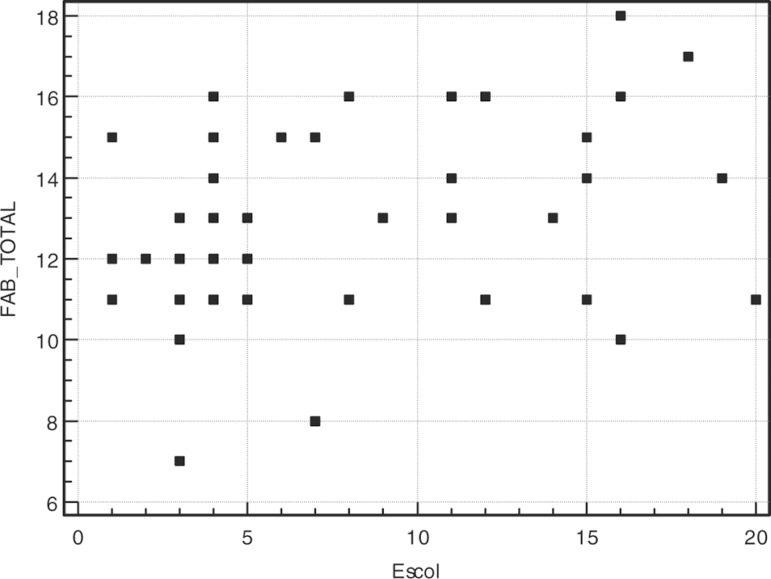
Correlation between FAB total score and education.

The mean total score ±SD of the FAB was 13.0±2.3, ranging from 7 to 18. The mean score ±SD of the MMSE was 27.4±1.8. Total FAB scores correlated significantly with educational level and with scores of the MMSE. No correlation was found between total scores of FAB and age. A separate analysis of each subtest of the FAB showed that only the subtests “Similarities” and “Conflicting Instructions” significantly correlated with educational level. The performance on the FAB and its correlation with the MMSE and educational level are presented in Graphs 1 and 2. Complete results of the statistical analysis are presented in Table 1. Administration of the FAB took less than 10 minutes.

**Table 1:** Summary of correlations found for the FAB.

Correlations	r	p
Total score X MMSE	**0.458**	**0.001**
Total score x Age	0.102	0.490
Total score X Education	**0.366**	**0.011**
Similarities x Education	**0.332**	**0.021**
Lexical fluency x Education	0.242	0.098
Motor programming x Education	0.128	0.385
Conflicting instructions x Education	**0.287**	**0.048**
Go / No-go x Education	0.201	0.170
Prehension behavior x Education	0.000	1.0

## Discussion

In the present study the FAB was administered to a group of elderly subjects, with no signs of cognitive impairment or depression. The FAB proved to be an easy test to administer, taking less than 10 minutes in the study sample.

Performance on the FAB, as expected, was influenced by educational level, as shown by the significant correlations found between total FAB scores and years of formal education. In addition, two subtests of the battery (“Similarities” and “Conflicting Instructions”) also correlated significantly with education. According to previous arti-cles, the item “Similarities” is largely influenced by education^33,34^. Similarly, the item “Conflicting Instructions”, which evaluates inhibition, is also influenced by educational level^35,36^. Surprisingly, no significant correlation was observed between the subtest “Lexical Fluency” and schooling, although there was a trend towards statistical significance (p=0.0979). It is well recognized that performance in verbal fluency tasks is heavily influenced by education^37,38^. Therefore, it is likely that the examination of a larger sample of individuals might reveal a similar feature in the letter fluency task of the FAB. This work is currently ongoing in our unit.

In a previous study we have observed an association between the performance of the subtest “Motor Programming” (or the Fist-Edge-Palm task of Luria) and education^39^. However, the same relationship has not occurred in the present study, which may reflect the larger number of individuals evaluated in the previous investigation as well as the inclusion of illiterates, most of whom had great difficulties performing the task.

We have found a significant association between the performance on the FAB and on the MMSE, in contrast to results reported by Dubois et al.^2^. These results are somewhat unexpected, since the MMSE does not formally evaluate executive functions. A possible explanation for this finding is an interaction between education and the performance in the MMSE, with the former being associated with the FAB. Indeed, there was a highly significant correlation between MMSE scores and educational level (r=0.601, p<0.0001; data nor shown). Nonetheless, further studies will help to confirm this hypothesis.

In conclusion, the Brazilian version of the FAB was well understood by cognitively healthy elderly and may be a feasible instrument for brief assessment of executive functions in the clinical setting. Additional work is currently being undertaken in our unit, with a larger sample of controls and also including patients with dementia, in order to determine the diagnostic accuracy of the FAB in our milieu and also to determine cut-off scores as a function of educational level.
